# Integrated Person-Centered Health Care for All Women During Pregnancy: Implementing World Health Organization Recommendations on Antenatal Care for a Positive Pregnancy Experience

**DOI:** 10.9745/GHSP-D-17-00141

**Published:** 2017-06-27

**Authors:** Sarah de Masi, Maurice Bucagu, Özge Tunçalp, Juan Pablo Peña-Rosas, Theresa Lawrie, Olufemi T Oladapo, Metin Gülmezoglu

**Affiliations:** a UNDP/UNFPA/UNICEF/WHO/World Bank Special Programme of Research, Development and Research Training in Human Reproduction (HRP), Department of Reproductive Health and Research, World Health Organization, Geneva, Switzerland.; b Department of Maternal, Newborn, Child and Adolescent Health, World Health Organization, Geneva, Switzerland.; c Department of Nutrition for Health and Development, World Health Organization, Geneva, Switzerland.

## Abstract

The 2016 WHO guideline on routine antenatal care (ANC) recommends several health systems interventions to improve quality of care and increase use of services including:

- Midwife-led continuity of care throughout the antenatal, intrapartum, and postnatal periods
- Task shifting components of ANC, including promotion of health-related behaviors and distribution of nutrition supplements
- Recruitment and retention of health workers in rural and remote areas
- Community mobilization to improve communication and support to pregnant women
- Women-held case notes
- A model with a minimum of 8 antenatal care contacts

In 2015, United Nations member states adopted the Sustainable Development Goals (SDGs) in order to continue the momentum of the Millennium Development Goals and address a broader range of development issues. The World Health Organization (WHO) identified target 3.8, universal health coverage, as the key to achieving all other health-related SDGs.^1^ To that end, the Every Woman Every Child movement developed the *Global Strategy for Women's, Children's, and Adolescents' Health (2016–2030)*^2^ with the aim of ending all preventable deaths of women, children, and adolescents and ensuring their health and well-being. The strategy provides a framework for countries to achieve the highest attainable standards of health for all women, children, and adolescents to “Survive, Thrive and Transform.”^2^

Building on the goal of achieving universal health coverage, WHO developed a global strategy for people-centered and integrated health services,^3^ recommending that countries consciously consider the perspectives of individuals, families, and communities, and respond to their preferences and needs. Furthermore, “WHO envisions a world where every pregnant woman and newborn receives quality care throughout pregnancy, childbirth and the postnatal period,”^4^ underscoring that the provision of integrated high-quality antenatal care (ANC) services is a critical part of the global agenda of achieving equitable, people-centered universal health coverage.^5^^,^^6^

In November 2016, WHO issued a new global guideline on routine ANC: *WHO Recommendations on Antenatal Care for a Positive Pregnancy Experience*.^5^ The main focus of the guideline is the prioritization of person-centered health and well-being in accordance with a human rights-based approach. The guideline defines having a positive pregnancy experience as^5^:

maintaining physical and sociocultural normality, maintaining a healthy pregnancy for mother and baby (including preventing or treating risks, illness and death), having an effective transition to positive labour and birth, and achieving positive motherhood (including maternal self-esteem, competence and autonomy).

This is the first WHO guideline on routine ANC to consolidate components of the essential core package of services and care that women and adolescent girls should receive throughout the pregnancy period. It is comprehensive in the sense that alongside prevention, detection, and management of pregnancy-related conditions, it also includes recommendations on health promotion, nutritional interventions, and prevention of concurrent diseases, such as malaria, HIV, and tuberculosis. This integrated approach aims to provide pregnant women with a universal package of quality care that ensures effective evidence-based practices, along with timely and relevant information and support during pregnancy.

The main focus of WHO's new global guideline on routine ANC is the prioritization of integrated person-centered health and well-being.

Intended to inform health care policies and clinical protocols, the 2016 WHO ANC recommendations are based on the most current evidence available and will be updated as new evidence emerges. Because ANC is currently an underutilized platform for maternal and perinatal health in many settings, we expect that through the implementation of these recommendations within an integrated quality service delivery approach, ANC can be a driver for health systems strengthening. The ANC recommendations were deliberately designed to be adaptable and flexible so countries with different health care settings, burdens of disease, social and economic situations, and health systems structures could ensure that the needs of their populations are met. These factors will inform the adaptation of the 2016 ANC model in terms of the content of the service package and determining *who* provides the care, *where* the care is provided, and *how* the care is provided to meet pregnant women's needs.

The WHO ANC recommendations were deliberately designed to be adaptable and flexible to meet different countries' needs.

Recruitment and retention of staff is an important strategy to providing good-quality ANC services in rural and remote settings.

The new guideline adopts a health systems approach, recommending several interventions to improve the quality of care delivery, which should lead to increased utilization and quality of ANC services (Table). At the same time, the guideline also aims to improve communication with, and support for, pregnant women throughout the antenatal period. This commentary focuses on health systems-related recommendations that are essential to the WHO ANC guideline implementation in countries.

## DELIVERY OF ANTENATAL CARE SERVICES

Midwifery-led continuity of care is a model of providing ANC that has been proven to improve maternal and newborn outcomes.^10^ In this model, a known and trusted midwife or a small group of known midwives are the primary providers of ANC for healthy pregnant women. The midwife provides support for the woman throughout the antenatal, intrapartum, and postnatal periods, facilitating a healthy pregnancy and childbirth as well as healthy parenting practices. Because this model of care requires an adequate number of skilled practicing midwives to ensure the continuum and quality of ANC, policymakers need to ensure that a well-functioning midwifery program is in place before scaling up the implementation of this model. Group ANC, provided by qualified health care professionals, integrates the usual individual pregnancy health assessment with tailored group educational activities and peer support, and might be an effective and feasible option to complement services offering individual ANC; however, at this point, group ANC is recommended only in the context of research.

Women highly value continuity of care. In low-resource settings, where the health workforce is often disproportionate to population needs and midwifery care may not yet be sufficiently available, task shifting components of ANC delivery can be a feasible way of improving women's access to ANC services and continuity of care, while policymakers work toward midwifery-led care for all women. In such contexts, the new WHO ANC guideline recommends task shifting the promotion of health-related behaviors for maternal and newborn health to a broader range of cadres, including lay health workers, auxiliary nurses, nurses, midwives, and doctors. The health-related behaviors covered by the recommendations include birth preparedness and complication readiness, insecticide-treated bed net use, companionship in labor and childbirth, nutritional advice, nutritional supplements, HIV testing during pregnancy, exclusive breastfeeding, postnatal care and family planning, and immunization. Additionally, the distribution of recommended nutritional supplements and intermittent preventative treatment in pregnancy for malaria prevention can be delivered by auxiliary nurses, nurses, midwives, and doctors. It is important that services provided as part of community outreach, including health promotion activities by lay health workers, are recognized and integrated into the health care system and that the task shifting occurs within a team approach, where regulatory support is provided and the mandate of all health workers involved is clearly defined.

Recruitment and retention of staff poses a major challenge to providing good-quality ANC services in rural and remote settings. Therefore, policymakers are encouraged to consider educational, regulatory, financial, personal, and professional support interventions to recruit and retain qualified health workers in such areas. Providing a safe and good working environment, including appropriate equipment and supplies, supportive supervision and mentoring, and relevant legal support, can motivate qualified health workers to both accept and remain working in rural and hard-to-reach environments.

In rural settings with low access to health services, community mobilization through facilitated participatory learning and action cycles with women's groups is also recommended, where feasible, to improve maternal and newborn health in these settings. These groups give women the opportunity to build close relationships with other pregnant women, enhance their sense of empowerment, and discuss their pregnancy needs and strategies for accessing care. Packages of interventions that include household and community mobilization are recommended and should be implemented alongside health systems strengthening interventions. Although antenatal home visits by lay health workers might be a helpful component of these packages, they should not replace regular ANC contacts.

The WHO ANC guideline recommends that pregnant women carry their own case notes.

Women should be empowered to access their own health-related information; to that end, the ANC guideline recommends that pregnant women carry their own case notes. In many low- and middle-income countries, this is already common practice as these case notes are often the only medical records available. Qualitative evidence suggests that women are likely to favor carrying their own case notes, and that this practice improves the continuity, quality, and experience of care for pregnant women.^11^ However, careful consideration needs to be taken as to what information is included in the case notes, as certain sensitive information, such as HIV status, can cause stigma and discrimination if the information is not kept confidential.

The 2016 WHO ANC guideline recommends an increase in the number of ANC contacts between the woman and the health care provider from 4 in the previous model to a minimum of 8 contacts. This recommendation is based on evidence that a minimum target of 8 contacts is associated with fewer perinatal deaths and a more positive pregnancy experience for women.^12^ The word “contact” is purposely used for this new recommendation, as it implies a more active connection between the woman and the ANC provider that allows sufficient time for provision of individualized person-centered care, including psychosocial and emotional support, for the woman. The 2016 WHO ANC model recommends the first contact occurring within the first trimester/12 weeks of gestation; 2 contacts in the second trimester, at 20 and 26 weeks; and 5 contacts in the third trimester, at 30, 34, 36, 38, and 40 weeks. The reason for increasing the number of contacts during the third trimester is that this is the period when the mother and unborn fetus are most at risk. Through increased contact many asymptomatic complications, such as hypertension, can be detected and managed, resulting in improved pregnancy outcomes. While all of the recommendations have been mapped to the 8 contacts in order to facilitate implementation of the guideline, countries still have considerable flexibility to determine how best to implement the recommendations and adapt the schedule to include practices and interventions best suited for their country context. These 8 contacts also provide enhanced opportunities for continuity of care for pregnant women and increased communication and trust within the woman–provider relationship. Quality of care is paramount, as evidence shows that if the quality of ANC is inadequate women do not attend ANC services regardless of recommended number of contacts in the model and, as a result, health benefits are reduced.

A minimum target of 8 ANC contacts is associated with fewer perinatal deaths and a more positive pregnancy experience for women.

## IMPLEMENTING THE NEW RECOMMENDATIONS

The integrated approach recommended in the new guideline gives countries the opportunity to use the ANC platform more effectively and efficiently, by providing comprehensive access to other relevant services, including those related to HIV, malaria, and tuberculosis. While the implementation of nationwide ANC programs in settings where health systems are already overstretched will be a challenge, investment in good-quality integrated ANC services in low- and middle-income countries will have far-reaching benefits for individual women, families, communities, and countries.

Investment in building the capacity of health care providers and increasing the number of midwives is absolutely imperative for improving the quality of ANC delivery and service utilization. Empowered health care professionals with excellent clinical and interpersonal skills are needed to provide quality ANC services. Therefore, staff pre- and in-service training programs should not only focus on evidence-based practices but also emphasize the need for positive staff behavior and attitudes. By receiving training in communication and respectful care, providers would have the necessary interpersonal skills to deliver individualized person-centered care. This will ultimately play a crucial role in the successful implementation of the ANC guideline.

In the context of a universal health coverage framework, the extent to which the new ANC guidelines will be adopted and scaled up will depend on nationally driven processes for the implementation and financing of comprehensive country ANC programs—under strong government leadership in long-term partnerships with key stakeholders. As recommended by WHO, countries should raise sufficient funds to support scale up, reduce reliance on direct payments from users to finance services, and improve the efficiency and equity of ANC services. Equally important to the implementation process is the active involvement of national professional organizations, end-users such as health care professionals, women's organizations, and community representatives to further increase chances of program sustainability.

WHO and partners are currently developing a set of policy briefs and an implementation guide for facilitating the adoption and adaptation of the *WHO Recommendations on Antenatal Care for a Positive Pregnancy Experience*.^5^ Regional dissemination of the new ANC guideline is underway in various WHO regions in partnership with Ministries of Health, other United Nations agencies, The Global Fund to Fight AIDS, Malaria and Tuberculosis, and other partner and professional organizations. WHO will continue to work with countries and partners to support the development of tools for implementation, monitoring, and evaluation of the WHO ANC model.

**TABLE.** Health Systems Interventions to Improve the Use and Quality of Antenatal Care as Recommended by the 2016 WHO Guideline on Routine Antenatal Care^5^

Recommended Interventions	Description
Midwife-led continuity of care	Midwife-led continuity-of-care models, in which a known midwife or small group of known midwives supports a woman throughout the antenatal, intrapartum, and postnatal continuum, are recommended for pregnant women in settings with well-functioning midwifery programs.
Group antenatal care	Group antenatal care provided by qualified health care professionals may be offered as an alternative to individual antenatal care for pregnant women in the context of rigorous research, depending on a woman's preferences and provided that the infrastructure and resources for delivery of group antenatal care are available.
Task shifting components of antenatal care delivery^a^	Task shifting the promotion of health-related behaviors for maternal and newborn health^b^ to a broad range of cadres, including lay health workers, auxiliary nurses, nurses, midwives, and doctors, is recommended.
	Task shifting the distribution of recommended nutritional supplements and intermittent preventive treatment in pregnancy for malaria prevention to a broad range of cadres, including auxiliary nurses, nurses, midwives, and doctors, is recommended.
Recruitment and retention of staff in rural and remote areas^c^	Policymakers should consider educational, regulatory, financial, and personal and professional support interventions to recruit and retain qualified health workers in rural and remote areas.
Community-based interventions to improve communication and support	The implementation of community mobilization through facilitated participatory learning and action cycles with women's groups is recommended to improve maternal and newborn health, particularly in rural settings with low access to health services.^d^ Participatory women's groups represent an opportunity for women to discuss their needs during pregnancy, including barriers to reaching care, and to increase support to pregnant women.
	Packages of interventions that include household and community mobilization and antenatal home visits are recommended to improve antenatal care utilization and perinatal health outcomes, particularly in rural settings with low access to health services.
Women-held case notes	It is recommended that each pregnant woman carries her own case notes during pregnancy to improve continuity, quality of care, and her pregnancy experience.
Antenatal care contact schedules	Antenatal care models with a minimum of 8 contacts are recommended to reduce perinatal mortality and improve women's experience of care.

a Recommendations adapted and integrated from WHO (2012).^7^

b Including promotion of the following: care-seeking behavior and antenatal care utilization, birth preparedness and complication readiness, sleeping under insecticide-treated bed nets, skilled care for childbirth, companionship in labor and childbirth, nutritional advice, nutritional supplements, other context-specific supplements and interventions, HIV testing during pregnancy, exclusive breastfeeding, postnatal care and family planning, and immunization according to national guidelines.

c Recommendation adapted and integrated from WHO (2010).^8^

d Integrated from WHO (2014).^9^

## Notes

**Peer Reviewed**
